# Intrinsic tumor factors and extrinsic environmental and social exposures contribute to endometrial cancer recurrence patterns

**DOI:** 10.21203/rs.3.rs-8682460/v1

**Published:** 2026-01-30

**Authors:** Jesus Gonzalez Bosquet, Oyomoare Osazuwa-Peters, Vincent M. Wagner, Andrew Polio, Rebecca Hoyd, Ahmad A. Tarhini, Casey M. Cosgrove, Marilyn S. Huang, Bradley R. Corr, Aliza L. Leiser, Bodour Salhia, Kathleen Darcy, Rob L. Dood, Lauren E. Dockery, Michael J. Cavnar, Lisa Landrum, Laura Chambers, Aik Choon Tan, Ning Jin, Robert J. Rounbehler, Michelle L. Churchman, Dan Spakowicz

**Affiliations:** University of Iowa; Duke University; University of Iowa; University of Iowa; The Ohio State University; Moffitt Cancer Center; The Ohio State University; University of Virginia; University of Colorado; Rutgers, The State University of New Jersey; University of Southern California; Walter Reed National Military Medical Center; Huntsman Cancer Institute; University of Oklahoma; University of Kentucky; Indiana University; The Ohio State University; Huntsman Cancer Institute; The Ohio State University; Aster Insights; Aster Insights; The Ohio State University

## Abstract

**Purpose:**

In a previous study, we trained, validated and tested models of endometrial cancer (EC) recurrence integrating clinical, genomic and pathological data from the Oncology Research Information Exchange Network (ORIEN). Preliminary studies also have demonstrated that bacterial communities may influence the risk of EC recurrence by altering the local environment within the upper female genital tract. The objective of this study was to evaluate whether extrinsic and environmental factors, including tumor-associated bacterial communities, tumor immune contexture and air pollution alongside clinical, pathologic and genomic features are associated with EC recurrence across clinically relevant risk groups.

**Patients and Methods:**

We performed a retrospective, multi-institution, case–control study with data from the ORIEN network EC dataset. Data was stratified into low-risk, FIGO grade 1 and 2, stage I (N = 329), high-risk, or FIGO grade 3 or stages II-IV (N = 324), and non-endometrioid histology (N = 239) groups. RNA and DNA were extracted from tumor specimens and processed to obtain the necessary genomic/metagenomic data. Genus level microbiome data were extracted and curated) from RNA sequencing using *Kraken2*, *Bracken* and *exotic* software packages. Risk of EC recurrence was evaluated by integrating microbiome and environmental data alongside existing clinical, pathological and genomic data using topic modelling with latent dirichlet allocation (LDA). Prediction models of EC recurrence were created using machine and deep learning analytics (ML and DL) with *MATLAB* apps and *TensorFlow*. Finally, performance of both topic and prediction models were externally validated in an independent EC dataset from TCGA.

**Results:**

The resulting models, analyzed with topic modelling, demonstrated the complexity of factors involved in recurrence of disease for EC. The components of the resulting topic models, and specifically the microbiome, changed when environmental factors, like air pollutants, were introduced in the model. In the low-risk EC group, microbes that were quite abundant in models before introducing environmental factors, were scarcely seen afterwards, like genera *Thermothielavioides*, *Theileria*, *Rhizoctonia*. *Bacillus* was the genus with higher per-topic probability within all risk groups, especially for low-risk EC (28%). Ozone (O_3_) was a resulting component of all risk groups’ models. BMI was the sole informative clinical variable after data integration, and only present in the low-risk group. Resulting models from the high-risk and non-endometrioid groups included differential gene expressions: *MMP13, S100A7, SMOC1, ACACA* and *ADD2, DLX5, SLCO2B1, NWD1* respectively. CNVs also were present in both low-risk and non-endometrioid groups, but their per-topic probabilities were low. The same was true for the immune contexture data. The components of the resulting topic models were used to train, validate and test prediction models of EC recurrence by risk groups. Performances of these models were excellent (@ 0.9). Despite some missing microbiome data in TCGA from resulting topic models, prediction models trained in the ORIEN set, had similar performances in TCGA testing set, with overlapping AUC 95% CIs.

**Conclusion:**

Both extrinsic factors (tumor-associated bacterial communities, tumor immune contexture and air pollution) and intrinsic factors predict EC recurrence. The complexity of tumor and host factors influencing cancer relapses underscore the need for more individualized prediction models of disease outcomes.

## BACKGROUND

The incidence and mortality for endometrial cancer (EC) continues to rise^1^ with a projected mortality increase of 55% by 2030.^2^ These discouraging outcomes are in part to the persistence of treatment failures, despite the recent introduction of immunotherapy and targeting therapies for this disease with notable successes (RUBY, GY-018, DUO-E).^3–5^ Though non-endometrioid EC types account for a disproportionately high number of EC recurrences and cancer-related deaths,^6^ the majority of treatment failures and recurrences occur in endometrioid EC, with approximately 10–15% of disease recurrence in patients with early-stage EC.^6,7^

In a previous study, we trained, validated and tested models of EC recurrence integrating clinical, genomic and pathological data from the Oncology Research Information Exchange Network (ORIEN).^8^ The models were stratified into low risk, FIGO grade 1 and 2, stage I (N = 329), high risk, or FIGO grade 3 or stages II, III, IV (N = 324), and non-endometrioid histology (N = 239) groups. This study resulted in validated high-performing prediction models, with area under the curve (AUC) performance over 0.9–0.95 for all 3 risk groups. While these models demonstrated excellent discrimination, they may not fully capture the biological complexity and environmental heterogeneity that influence EC recurrence across diverse patient populations. To further improve discriminatory accuracy and generality of these models, we hypothesized that inclusion of intrinsic tumor microenvironmental (TME) variables and extrinsic environmental variables alongside clinical, pathologic and genomic features may modify geographically the risk for EC recurrence.

In preliminary data, we observed that the microbiome is associated with female genital tract cancers, specifically with EC, and may interact differently with tumors with different mutation signatures.^9,10^ The human microbiome is a symbiotic community of bacteria, fungi, and viruses that live on or within the human body with specific functions, properties, and interactions within its environment.^11,12^ Bacterial communities may influence the risk of EC recurrence by altering the local immune response modulation, by epigenetic changes, or TME modulation.^13–15^ Additionally, other environmental factors, like air pollution also have been associated to incidence and recurrence in hormonal-related cancers, like breast cancer,^16^ or acting as xenoestrogens or anti-androgens, inducing oxidative stress, DNA damage, epigenetic changes, and chronic inflammation in hormone-sensitive tissues.^17,18^

The objective of this study was to assess the differences in EC recurrence risk when accounting for TME factors, like tumor-associated microbiome and immune cell infiltration, and extrinsic environmental factors, like air pollution and environmental determinants of health. Then, we assessed the predictive accuracy of these intrinsic and extrinsic variables for EC recurrence. Performance of the prediction models were externally validated using the Cancer Genome Atlas (TCGA) EC datasets.

## METHODS

### Study design:

We performed a retrospective, multi-institution, case–control study with data originated from the ORIEN network EC dataset. ORIEN is comprised of multiple cancer centers that have agreed to use the same Institutional Review Board (IRB)-approved protocol and consent (Total Cancer Care Protocol, TCC) to follow patients throughout their lifetime.^19^ A copy of the protocol is included in **Supplementary Material**. Patients consent to donate medical records and tissue specimens for molecular profiling, as an approach to improve design and performance of personalized cancer care. RNA and DNA were extracted from tumor specimens and processed to obtain the necessary genomic/metagenomic data, as specified previously.^8^ The study analysis was carried out in several steps: 1) *Step 1*: selection of models and variables included in the preliminary study of EC prediction of recurrence that included clinical, genomic and pathologic data;^8^ 2) *Step 2*: extraction and curating of microbiome data (at the taxa level of genus) from RNA sequencing (RNAseq) experiments using *Kraken2*, *Bracken* and *exotic* software packages; 3) *Step 3*: using topic modelling, as described previously,^20^ to determine microbiomes (genus taxa) associated with EC recurrence by risk groups; 4) *Step 4*: determine social and environmental determinants of health associated with EC recurrence; 5) *Step 5*: integration of significant genomic, microbiome and environmental factors (resulting from previous steps) with topic modelling, to identify those factors associated with EC recurrence by risk groups; 6) *Step 6*: assessment of how these variables from significant topics associated with EC recurrence performed as prediction models of recurrence using machine and deep learning analytics (ML and DL) with *MATLAB* apps and *TensorFlow*. Finally, these steps with integration of elements of EC recurrence and EC prediction modelling were externally tested (validated) in an independent EC dataset, TCGA.

### Patients’ inclusion, clinical, pathological and genomic data:

Details of patients inclusion in risk groups, clinical and pathological data included and genomic data extraction, processing and analysis (Step 1 of the study design) are detailed in a previous publication.^8^ Briefly, we included all patients in ORIEN database with EC, including all histologies, that had information about recurrent disease. Patients with EC recurrence (or **cases**) were those that after completion of treatment with no evidence of disease (NED), EC reappeared, either locally (vaginal), regionally (pelvis) or distally. Index cases included women with a new event of EC cancer after treatment, those who had cancer at the last surveillance or died from cancer. **Controls** were patients with NED during the whole follow-up. There was a total 892 women with EC included in this analysis: 186 with EC recurrence (cases) and 706 without (controls), that had RNA and DNA sequenced and had recurrence information (**Supplementary Table 1**, also in Gonzalez Bosquet J., et al.^8^). Included patients were part of ORIEN database since 2004 and up to 2021. Patients with 2009 FIGO stage I and histological grade 1 or 2 endometrioid EC had an overall recurrence rate of 11.6% (38/329) and were considered low risk for recurrence. Patients with a histological grade 3 endometrioid EC or with FIGO stage II-IV had an overall recurrence rate of 21.3% (69/324) and were considered high-risk for recurrence. Patients with non-endometrioid type EC (serous, carcinosarcoma, clear cell, undifferentiated, mixed) had an overall recurrence rate of 33.1% (79/239) and have even higher risk for recurrence.

Baseline variables were collected after surgery, when histologic type, FIGO stage and other clinical and demographic characteristics were known. Resulting models and variables included in the preliminary study of EC recurrence prediction, which included clinical, genomic and pathologic data,^8^ were selected and incorporated into the integrated dataset to be analyzed with topic modelling (**Supplementary Table 2**, also in Gonzalez Bosquet J., et al.^8^).

### Tumor microenvironment (TME) data:

Microbiome data. *Data preprocessing*: CRAM files were downloaded from the Orien server and then converted into BAM files with *samtools* for further analysis. Analyses were performed as outlined by the NCI Genomic Data Commons (GDC - https://docs.gdc.cancer.gov/Data/Introduction/). The *STAR* suite (including *STAR-Fusion*) were used to align the transcriptome to the genome assembly version CHM13 T2T.^21,22^ We used the *exotic* pipeline to broadly but conservatively identify microbes present in the tumors while removing technical artifacts and contaminants from the dataset (Step 2 of the study design).^23^ First, exotic maps raw reads with quality scores (FASTQs) to the human reference genome, with a second alignment pass following the standard workflow of TCGA and other large-scale sequencing efforts. Next, *exotic* aligns the unmapped reads to a wide range of non-human genomes, including bacteria, archaea, viruses, fungi, and a subset of other eukaryotes using the *KrakenUniq* option from the *Kraken2* pipeline.^24^ Then, it uses *Bracken* for estimation of abundance at a the genus taxa level using the resulting classification from *KrakenUniq*.^25^ Next, *exotic* filters contaminants in two phases: statistical filtering and literature matching.^23^ Finally, the outputs are normalized to remove technical artifacts. In summary, exotic discards a small fraction of the reads in the statistical filtering step, though these reads represent a large fraction of the total microbes; and removes a large fraction of the reads but relatively few taxa with the literature-based filtering.

#### Data analysis:

Topic modeling with Latent Dirichlet Allocation (LDA)^26,27^ was used to assess changes in microbial communities between samples (Step 3 of the study design): 1) first, *Idatuning* method determined the optimal number of latent topics for the analysis; 2) then, we used *Topicmodels* to evaluate differences in microbial communities by examining topic distributions (both R packages).^28^ Statistical differences between the two groups (controls vs cases) were considered for false discovery rate (FDR) adjusted p-values < 0.05. The use of topic modeling (as natural language processing – NLP) allows to assess how microbiome communities differ quantifiably and in their composition. By treating microbial communities as “topics”, like how words cluster in textual data, we were able to model the high-dimensional interactions between different genus and identify potentially meaningful patterns and associations EC recurrence.

Again, variables (genus) included in the resulting models were selected and incorporated into the integrated dataset to be analyzed with topic modelling.

Tumor immune environment. To evaluate the tumor micro-environment and the immunity response induced by the tumor, we assessed the immune contexture (or the type of tumor-infiltrating immune cells)^29^ and the cancer associated fibroblasts (or CAF). This evaluation could be very informative of the types of inflammatory, angiogenic, and desmoplastic reactions occurring in a tumor.

We measured the immune contexture with *quanTIseq*, a computational pipeline that uses bulk RNAseq data using a novel deconvolution approach.^29^ We used RNAseq resulting from previous steps.

We used the Microenvironment Cell Populations (MCP)-counter, a transcriptome-based computational method that quantifies the abundance of tissue-infiltrating immune and non-immune stromal cell populations in non-hematopoietic human tumors.^30^ This method also uses the gene expression matrix resulting from RNAseq to determine the abundance score for CD3 + T cells, CD8 + T cells, cytotoxic lymphocytes, NK cells, B lymphocytes, cells originating from monocytes (monocytic lineage), myeloid dendritic cells, neutrophils, as well as endothelial cells and fibroblasts.

### Social and Environmental data:

Environmental variables. Air pollution data for year 2010 was obtained at the county level for four gases (O_3_, CO, SO_2_, NO_2_), and two aerosols (PM_10_, PM_2.5_), from the Center for Air, Climate and Energy Solutions (CACES; https://www.caces.us/data). This air pollution data was linked with ORIEN data for EC study cohort by Aster Insights collaborators who handle data pull by cross-referencing unique county identifiers in the air pollution data with five-digit zip codes for each patient in the EC study cohort. Air pollution data for each eligible patient was provided with the county code only, to prevent identification of individual patients. Not all patients had information from the county code.

Social and environmental determinants of health. Social and environmental determinants of health were derived from the Centers for Disease Control and Prevention’s Environmental Justice Index (EJI) dataset. The EJI is a nationwide, place-based index designed to capture cumulative health impacts from environmental and social burdens at the census tract level. It comprises 36 indicators organized into 10 domains—Racial/Ethnic Minority Status, Socioeconomic Status, Household Characteristics, Housing Type, Air Pollution, Potentially Hazardous and Toxic Sites, Built Environment, Transportation Infrastructure, Water Pollution, and Preexisting Chronic Disease Burden—and grouped into three overarching modules: Social Vulnerability, Environmental Burden, and Health Vulnerability.

For this study, we extracted the percentile rank scores for each of the 10 domains from the EJI dataset, which was downloaded from the Agency for Toxic Substances and Disease Registry website. In addition, food access data were obtained from the United State Department of Agriculture’s Food Access Research Atlas, specifically the variable low access tract at 1 mile for urban areas or 10 miles for rural areas. This variable is defined as “a low-income tract with at least 500 people or 33% of the population living more than 1 mile (urban) or more than 10 miles (rural) from the nearest supermarket, supercenter, or large grocery store.” These census tract–level social and environmental determinants were linked to patient-level data using the county code corresponding to each census tract as the unique identifier. For counties containing multiple census tracts, data were summarized using a weighted mean, with weights based on census tract population size. Additional details on all variables used to capture social and environmental determinants of health are provided in **Supplementary Table 3**.

Data analysis. We used bipartite network analysis to identify clusters (subtypes) of both patients and social and environmental determinants of health. Bipartite network takes input data at the county-code level and outputs a quantitative summary (number, size, and statistical significance) along with a network visualization of the identified clusters.^31^ Statistical significance was assessed by comparing the observed value to a null distribution generated from 1,000 random permutations of the network.^32^ Compared to traditional clustering methods such as hierarchical clustering or principal component analysis, bipartite networks offer two key advantages: (1) they operate autonomously without requiring user-defined parameters, and (2) they define clusters that include both patients and variables.^32^ We used bipartite networks to detect clusters and associations between cluster membership and recurrence of disease between air pollution and social determinants of health. Bipartite network separated air pollution data and social determinants of health, so we performed a multivariate *lasso* regression of EC recurrence for both domains, selected those variables that were most informative for EC recurrence prediction for both, and then, selected variables from both domains, were incorporated into the integrated dataset to be analyzed separately with topic modelling.

### Integration of resulting models:

All elements significant in the clinical, pathological, genomic, microbiological, and environmental analyses were added to integrated databases and analyzed with topic modelling to assess patterns and associations between different data types and EC recurrence (Step 4 of the study design). Because environmental and social variables were less available in the dataset, and separated by bipartite networks, models were performed with and without them.

#### Training, validating and testing EC recurrence models:

Finally, we trained, validated and tested models with the integrated datasets that included all selected variables resulting from topic modelling (Step 5 of the study design). For prediction modelling we used *lasso* regression, other machine learning (ML) included in *MATLAB* apps, and deep learning (DL) with *TensorFlow* analytics. Briefly, for *MATLAB* analysis, we used 10-fold cross-validation for training, and left 20% of EC samples for testing with, using 35 ML different methods on ORIEN dataset. The best models were selected for reporting. Model explanation was performed on training and testing models using Shapley values.^33^ In the context of machine learning prediction, the Shapley value of a feature for a query point explains the contribution of the feature to a prediction (score of each class for classification) at the specified query point. We use the Shapley values of predictors to interpret which predictors have the largest (or smallest) average impact on model output magnitude.

Additionally, we used *TensorFlow*^34^ in a *Jupyter* notebook with a *Keras* application programming interface (API)^35^ as the DL method. This is a modification of the *TensorFlow* core tutorial ‘Classification of imbalanced data’ (www.tensorflow.org/tutorials/structured_data/imbalanced_data). Normalization of the data was performed using the *sklearn StandardScaler*. Models had 16 layers, with a dropout layer to reduce overfitting, and an output sigmoid layer that returns the probability of a transaction being fraudulent. The input layer of each model contained as many nodes as features to analyze. Training was performed to account for weights of the outcomes as well as for unbalanced data using oversampling methods. Validation was done using 15% of samples and 25% of samples were kept for testing the models.

### Validation in TCGA EC data:

Data preprocessing. TCGA BAM files initially were converted to FASTQ files with the *samtools* pipeline. Then, the rest of the genomic and microbiome extraction was performed as detailed in the ORIEN database.

Validation analysis. Validation was performed using TCGA EC dataset, that included endometrioid and serous EC (TCGA-UCEC)^8^ and endometrial carcinosarcoma (TCGA-UCS). Briefly, after permission was granted to access controlled data by the Genomic Data Commons (GDC) Data Portal (dbGaP#29868), TCGA-UCEC RNAseq (406 endometroid and 136 serous EC) and TCGA-UCS RNAseq (56 endometrial carcinosarcomas) files in BAM format were downloaded from women with EC. Main clinical characteristics are described in **Supplementary Table 4**. Of note is that non-endometrioid cases in TCGA did not include any clear cell, undifferentiated, or dedifferentiated carcinomas. For validation we used only those significant variables resulting from topic modelling that were selected and included in the integrated dataset (Step 4 of the study design). We used TCGA dataset first to externally validate the models associated with EC recurrence that included clinical, genomic and microbiome data. County codes were not available for TCGA patients, so we were not able to link all metagenomic/genomic data with environmental and socials determinants of health. Additionally, we used TCGA datasets for external testing of the best prediction models for EC recurrence trained in the ORIEN set. The best prediction models of EC recurrence were tested with ML learning (*MATLAB*) and with DL (*TensorFlow*) and including TCGA data as the testing set. Survival analysis prediction with Cox proportional hazard ratios and Kaplan-Meir survival curves were performed in R with *survival* and *ggsurvfit* packages.

## RESULTS

### Tumor-associated microbiome communities associated with EC recurrence:

**Figure 1:**
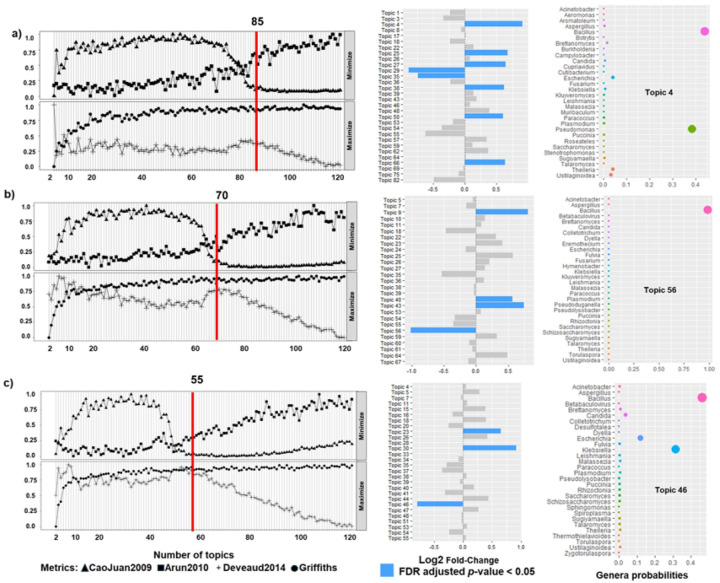
**Topic modelling for microbiome communities: Analysis to identify an optimal and significant topics in EC (left panels):** We utilized the *FindTopicNumber* function from the ***ldatuning***package to identify an optimal latent topic number for our model based on 4 different metrics: minimization for Arun2010 and CaoJuan2009, and maximization for Deveaud2014 and Griffiths2004. For minimization metrics a lower value suggests an optimal topic structure; and for maximization metrics a higher value suggests an optimal topic structure. Optimal topic numbers are represented in left panels: **a)** Low risk EC; **b)** High risk EC; **c)**Non-endometrioid EC. **Selecting topics via Latent Dirichlet Allocation (LDA – Middle and Right panels):** LDA is popular for natural language processing for topic modeling. LDA computes differential abundance analysis, to identify differentially abundant topics between recurrent and non-recurrent cohorts (middle panels). Selected in blue are those topics with log2 fold changes and false discovery rate (FDR) corrected p-values < 0.05: **a)** Low risk EC, topics 4,25,27,29,35,38,50,66; **b)** High risk EC, topics 9,40,43,56; **c)**Non-endometrioid EC, topics 23,30,46. In the **right panel** we depict per-topic-species probabilities matrix to examine which genus have the highest probabilities of assignment to this topic/community: **a)** Low risk EC, topic #4; **b)** High risk EC, topic 56; **c)** Non-endometrioid EC, topic 46.

First we identified the optimal number of latent topics for each EC recurrence risks groups: low risk (85 latent topics), high risk (70 latent topics) and non-endometrioid group (55 latent topics) (Figure 1, left panels). Then, we used latent Dirichlet allocation (LDA) to identify differentially abundant topics by comparing topic distributions profiles between recurrence groups (Figure 1, middle panels). Topics were considered statistically significant topics if they met an FDR corrected p-values threshold of < 0.05 and demonstrated negative log2 fold changes.

### Tumor micro-environment features associated with EC recurrence:

**Figure 2:**
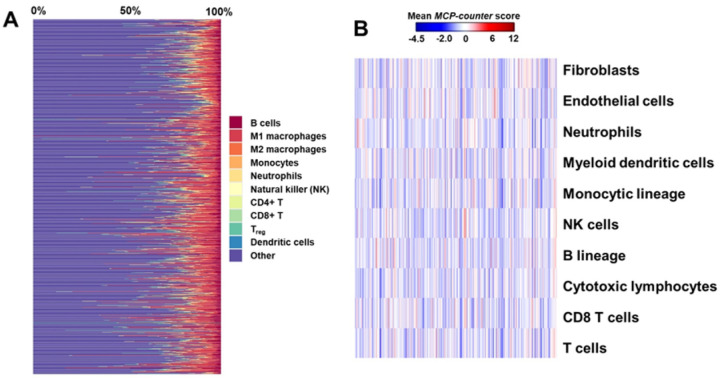
Deconvolution of bulk RNA to assess immune contexture and cancer associated fibroblasts (CAF). **A**. Deconvolution of bulk RNAseq from ORIEN EC sample set with *quanTIseq*, a computational pipeline for the quantification of the Tumor Immune contexture: type and density of tumor-infiltrating immune cells. from human RNA-seq data. The proportion of immune cells infiltrating the tumor was 26% in average (range 2–100%). Each type of resulting immune cell is color coded in the annotation side panel: B cells, M1 and M2 macrophages, monocytes, neutrophils, natural killer (NK) cells, non-regulatory CD4+ T cells, CD8+ T cells, T_reg_ cells, and myeloid dendritic cells (DC). **B**. Heatmap of Microenvironment Cell Populations (MCP) counter score, using the log2 geometric mean of this set of markers for each immunologic cell. Samples (columns) are order by tissue type: HGSC or normal tube; and the cell type (rows) are: CD3+ T cells, CD8+ T cells, cytotoxic lymphocytes, NK cells, B lymphocytes, cells originating from monocytes (monocytic lineage), and myeloid dendritic cells, and neutrophils, endothelial cells and fibroblasts.

We assessed the tumor immune microenvironment and CAF using gene expression patterns derived from RNAseq (Figure 2). Topic modelling was applied to determine which of these cellular components were most informative for EC recurrence. Immune cell populations identified in this initial analysis were subsequently introduced into the integrated topic modeling framework alongside significant genomic, metagenomic and clinical features, stratified by risk group.

### Environmental data associated with EC recurrence:

**Figure 3:**
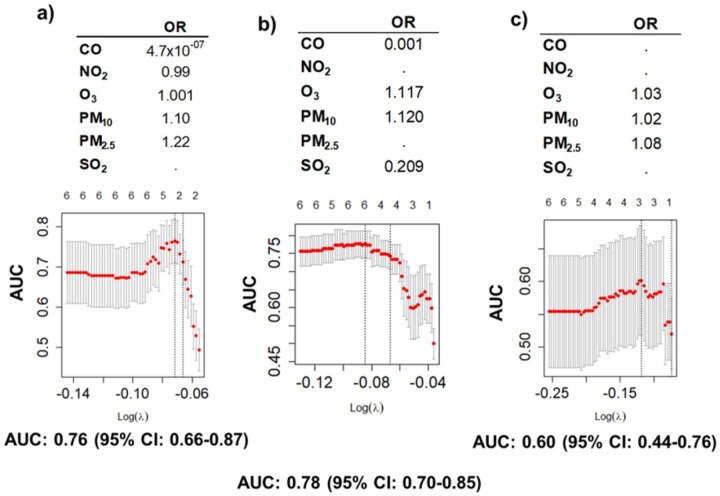
Air pollutants by county code. We performed a multivariate lasso regression analysis to identify which air pollutants were associated with EC recurrence. Those selected were integrated in the final analysis including all significant variables. **Upper panels** show tables with odd ratios (OR) of selected air pollutants resulting from the lasso regression analysis for EC recurrence: a) Low risk EC: NO_2_, O_3_, PM_10_, PM_2.5_; b) High risk EC: O_3_, PM_10_, SO_2_; c) Non-endometrioid EC: O_3_, PM_10_, PM_2.5_. **Lower panels** represent lasso multivariate regression results (glmnet R package): the upper axis represent the number of variables included in the model; the y axis is the performance of the model measured by AUC, with the 95% confidence interval (CI): a) Low risk EC: the best performance of the model is with 5 variables; b) High risk EC: best performance with 4 variables; c) Non-endometrioid EC: best performance with 3 variables. The lower axis is the log of the λ, value used to optimize model construction. We performed 1,000 bootstrap replicates to find the most adequate λ for the model. Underneath each graphic is the performance of the model by the AUC with the 95% CI.

For low-risk EC, five out of six air pollutants were informative for EC recurrence, including CO, NO_2_, O_3_, PM_10_, PM_2.5_; while for high-risk four out of six, and for non-endometrioid three out of six (Figure 3). Aerosols, PM_10_, PM_2.5_ consistently showed increased risk (OR > 1), like O_3_, while CO, NO_2_, and SO_2_ showed inverse associations (OR < 1). Variables selected by this model were then incorporated into the integrated datasets together with significant genomic, metagenomic and clinical data variables for the final analysis. Social and environmental determinants of health initial lasso regression are presented in **Supplementary Figure 1**.

### Integration of resulting models:

All features identified as significant across clinical, pathological, genomic, microbiological, and environmental analyses were incorporated to an integrated dataset and analyzed using topic modelling. Three integrated models were evaluated: a) a model including clinical, genomic, and immune features (Clin+Gen+Imm); b) a model additionally incorporating air pollution variables (Clin+Gen+Imm+Pol); and c) a model further including social and environmental determinants of health data (Clin+Gen+Imm+Env).

This stepwise modeling strategy was employed because county identifiers linking environmental data to patients were available for air pollution in 74% of cases and for social/environmental determinants of health in only 64% of patients.

**Figure 4:**
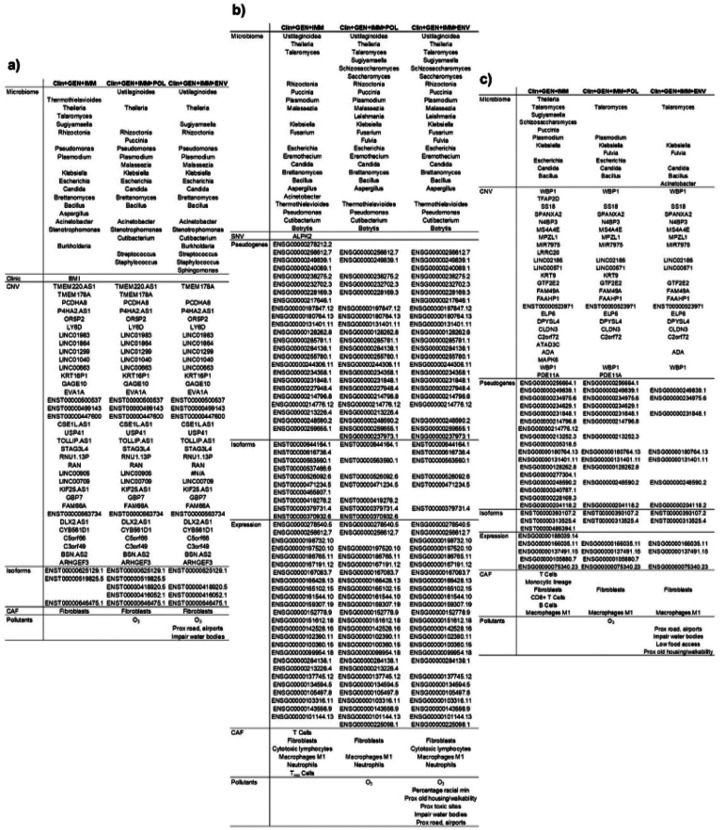
Resulting components of the significant topic models from integrated databases. For the 3 risk groups, low EC (a), high EC (b), and non-endometrioid EC (c), we represented the components resulting from the topic models analyses with/without environmental factors. In all 3 tables, at the left is the variable type, the first column are the components of the model without environmental variables and the next two with them: Clin+Gen+Imm: including clinical, genomic/metagenomic, immune contexture; Clin+Gen+Imm+Pol: including clinical, genomic/metagenomic, immune contexture, and air pollution. Clin+Gen+Imm+Env: including clinical, genomic/metagenomic, immune contexture, and all environmental factors.

The composition of significant topics across all three EC risk groups (low-risk, high-risk, and non-endometrioid) with and without environmental variables is summarized in Figure 4. Topic model optimization and computational performance for each integrated model (Clin+Gen+Imm, Clin+Gen+Imm+Pol, and Clin+Gen+Imm+Env) are presented in **Supplementary Figures 2, 3**, and 4, respectively.

Per-topic variable probabilities, detailing the expected average probability for each component within a given topic, indicated that *Bacillus* was the most probable microbial genus across all risk groups, especially for low-risk EC (28%) but also for non-endometrioid type (10%)(**Supplementary Table 5**). In addition, *Stenotrophomonas* (10%) and *Thermothielavioides* (27%) were frequently observed in significant recurrence-associated topics in low-risk EC.

Among clinical variables, BMI was the only feature retained after data integration; however, it was observed exclusively in the low-risk group and at a low probability (0.6%). Variables with higher per-topic probabilities (>10%) were predominantly gene expression features. In the high-risk group, these included ENSG00000137745.12 (*MMP13*), ENSG00000143556.9 (*S100A7*), ENSG00000198732.10 (*SMOC1*), and ENSG00000278540.5 (*ACACA*). In the non-endometrioid group, high probable genes included ENSG00000075340.23 (*ADD2*), ENSG00000105880.7 (*DLX5*), ENSG00000137491.15 (*SLCO2B1*), ENSG00000188039.14 (*NWD1*), along with pseudogenes ENSG00000128262.8 (*POM121L9P*), ENSG00000234975.6 (*FTH1P2*).

CNVs were detected in both low-risk and non-endometrioid groups; however, their probabilities within significant topics were consistently low. Similarly, immune contexture features, CAF, and gene isoforms expression contributed at low frequency. SNVs were infrequent and were not prominent in any risk group. Although air pollutants and social/environmental determinants of health were present across all models, their per-topic probabilities were uniformly low (<1%).

Notably, the inclusion of environmental variables altered the composition of microbiome-associated features within the resulting topic models, suggesting interactions between environmental exposures and tumor-associated microbial communities.

### Training, validation and testing models for EC recurrence:

We developed, validated and tested predictive models for EC recurrence using features from significant topics identified in the integrated dataset. This analysis evaluated whether topic-selected features were also informative predictors of recurrence. Models were trained and cross-validated using two analytical platforms: *MATLAB*-based machine learning (ML) and *TensorFlow*-based deep learning (DL). For MATLAB, only the best performing models were retained from 35 candidate configurations. For *TensorFlow*, training accounted for class imbalance through oversampling strategies, as recurrence events comprised approximately 10–30% of samples.

**Figure 5:**
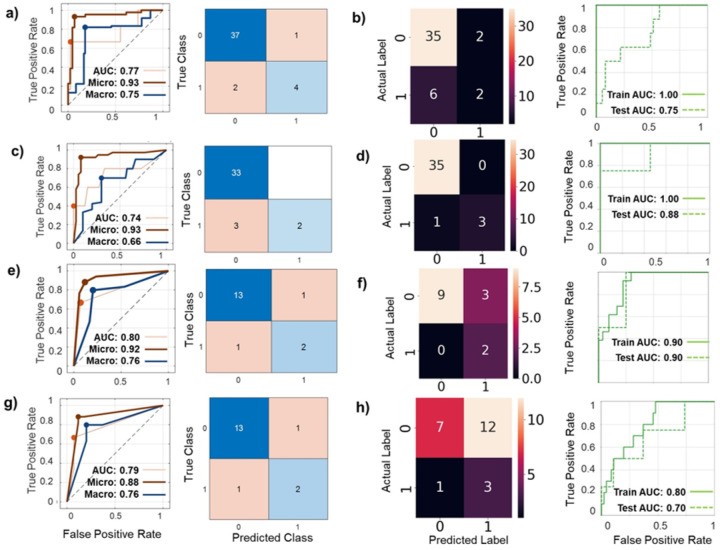
Training, validation and testing of prediction models of low-risk EC recurrence using components of significant topics from the LDA analyses done with machine learning (ML - *MatLab*) and with deep learning (DL - *TensorFlow*). **a)** Training, validation and testing of a low-risk EC recurrence model using MatLab (Ensemble, subspace discriminant) and components from the significant topics integrating clinic and microbiome data: the left panel shows the testing ROC curves with AUC, micro-average AUC and macro-average AUC. Microaverage takes imbalance into account in the sense that the resulting performance is based on the proportion of every class; the right panel shows the model testing confusion matrix. **b)** Training, validation and testing of a low-risk EC recurrence model using *TensorFlow* and components from the significant topics integrating clinic and microbiome data: the left panel shows the model testing confusion matrix; the right panel shows training and testing ROC curves with AUC. **c)** Training, validation and testing of a low-risk EC recurrence model using MatLab (Ensemble, subspace KNN) and components from the significant topics integrating clinic, microbiome, genomic and cell immunocompetent infiltration data: the left panel shows the testing ROC curves with AUC, micro-average AUC and macro-average AUC; the right panel shows the model testing confusion matrix. **d)** Training, validation and testing of a low-risk EC recurrence model using *TensorFlow* and components from the significant topics integrating clinic, microbiome, genomic and cell immunocompetent infiltration data: the left panel shows the model testing confusion matrix; the right panel shows training and testing ROC curves with AUC. **e)** Training, validation and testing of a low-risk EC recurrence model using MatLab (Fine tree) and components from the significant topics integrating clinic, microbiome, genomic, cell immunocompetent infiltration, and air pollution data: the left panel shows the testing ROC curves with AUC, micro-average AUC and macro-average AUC; the right panel shows the model testing confusion matrix. **f)** Training, validation and testing of a low-risk EC recurrence model using *TensorFlow* and components from the significant topics integrating clinic, microbiome, genomic, cell immunocompetent infiltration, and air pollution data: the left panel shows the model testing confusion matrix; the right panel shows training and testing ROC curves with AUC. **g)** Training, validation and testing of a low-risk EC recurrence model using MatLab (Binary GLM logistic regression) and components from the significant topics integrating clinic, microbiome, genomic, cell immunocompetent infiltration, and all social and environmental determinants of health data: the left panel shows the testing ROC curves with AUC, micro-average AUC and macro-average AUC; the right panel shows the model testing confusion matrix. **h)** Training, validation and testing of a low-risk EC recurrence model using *TensorFlow* and components from the significant topics integrating clinic, microbiome, genomic, cell immunocompetent infiltration, and all environmental data: the left panel shows the model testing confusion matrix; the right panel shows training and testing ROC curves with AUC.

Separate recurrence predictions models were trained for each risk group, low-risk EC (Figure 5), high-risk (**Supplementary Figure 5**) and non-endometrioid EC (**Supplementary Figure 6**). For each group we trained models including different combinations of data. Figure 5 summarizes model performance for the low-risk group, as measured by the area under the receiver operator characteristics curve (AUC), for models incorporating: clinical and metagenomic data (Clin+Gen; Figure 5a and 5b); clinical, microbiome, genomic and immune contexture (Clin+Gen+Imm; Figure 5c and 5d); clinical, microbiome, genomic, immune contexture, and air pollution data (Clin+Gen+Imm+Pol; Figure 5e and 5f); and clinical, microbiome, genomic, immune contexture, and social/environmental data (Clin+Gen+Imm+Env, Figure 5g and 5h). Equivalent modeling strategies were applied to the high-risk (**Supplementary Figure 5**) and non-endometrioid groups (**Supplementary Figure 6**).

Across all three risk groups and both analytical platforms, models containing clinical, microbiome, genomic and immune contexture features (Clin+Gen+Imm) demonstrated the strongest performance in the testing set. For low-risk EC, AUCs reached 0.93 using MATLAB (Figure 5c) and 0.88 using Tensorflow (Figure 5d). In high-risk EC, corresponding AUCs were 0.9 (MATLAB; **Supplementary Figure 5c**) and 0.85 (Tensorflow; **Supplementary Figure 5d**). In non-endometrioid EC, AUCs were 0.79 (MATLAB; **Supplementary Figure 6c**) and 0.76 (Tensorflow; **Supplementary Figure 6d**).

Although inclusion of environmental variable, air pollution (Clin+Gen+Imm+Pol; Figure 5e and 5f) and social/environmental determinants of health (Clin+Gen+Imm+Env, Figure 5g and 5h), reduced sample size due to missing county-level data (see confusion matrix in low-risk and non-endometrioid groups - **Supplementary Figure 6e-h**), model performance in testing sets remained acceptable. This was particularly evident in the high-risk group: where AUC reached 0.89 for Clin+Gen+Imm+Pol and 0.8 for Clin+Gen+Imm+Env models (**Supplementary Figure 5e-h**).

To assess the relative contribution of individual predictors within the best-performing models, we applied Shapley value analysis (**Supplementary Figure 7**). Incorporation of air pollution measures and social/environmental determinants of health consistently altered the ranking and composition of the most influential predictors across all three EC risk groups, with particularly pronounced effects on microbiome-associated features (**Supplementary Figure 7d-i**). These effects were most evident in the non-endometrioid group (**Supplementary Figure 7i**), where multiple social/environmental determinants, proximity to high volume roadways and airports, proximity to impaired water bodies, and limited food access, emerged as influential contributors to recurrence prediction.

### External testing of models for EC recurrence:

After downloading and pre-processing TCGA EC dataset using the same pipeline applied to the ORIEN cohort, we extracted variables corresponding to those retained in the integrated topic models encompassing clinical, genomic/metagenomic and immune context features. To first assess whether the EC risk group stratification derived from ORIEN was comparable in TCGA, we evaluated progression-free survival (PFS) across low-risk, high-risk, and non-endometrioid groups in both datasets (**Supplementary Figure 8**). Although differences in PFS were observed, the 95% CIs for all three risk groups overlapped substantially, particularly during the first 2–3 years of follow-up. TCGA represents a valuable external resource but had known limitations that may affect validation performance,^8^ including missing variables, limited follow-up and case status reporting, incompletely staged cases, and differences in timing of biospecimen collection. These factors are likely to contribute to the divergence of PFS curves observed later in follow-up.

County-level identifiers are not available in TCGA because they constitute personal identifying data, therefore, environmental exposures could not be linked to TCGA EC cases. In addition, several features present in the integrated ORIEN topic models were not available in TCGA: a) in low-risk EC 29% of significant components missing: CNVs (mainly in long non-coding RNAs) and some microbiomes, like the genus *Thermothielavioides* with a probability of 27% of being a component of the resulting topic, genus *Theileria* and *Rhizoctonia* with probabilities below 8%, and the rest with probabilities below 4%; b) in high-risk EC 18% of significant components missing: like genus *Malassezia* with a probability of 4% and *Candida* with a probability of 5%; the rest missing components had probabilities of 2% or below; c) the non-endometrioid group had only 11% of missing components all of them with probabilities below 0.5%. Notably, *Thermothielavioides* was absent from all resulting topic models after air pollution variables were introduced, while *Theileria*, *Rhizoctonia*, *Malassezia*, *Candida* appeared in only one of four topics when air pollution was included (Figure 4).

**Figure 6:**
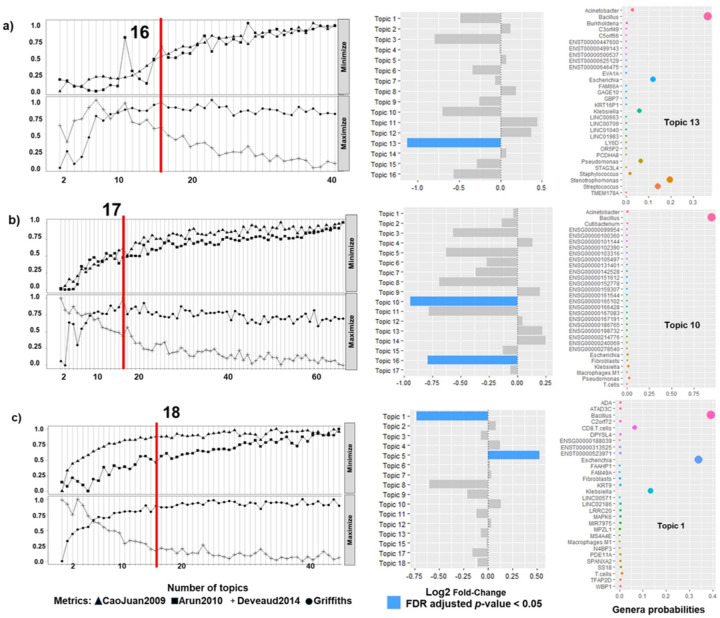
TCGA validation of integration of clinical, genome, bacteriome, immunocompetent cells proportions with topic modelling. Analysis to identify optimal and significant topics in EC (**left panels**): To identify an optimal latent topic number for our model based on 4 different metrics: minimization for Arun2010 and CaoJuan2009, and maximization for Deveaud2014 and Griffiths2004. Optimal topic numbers are represented in left panels: **a)** Low risk EC; **b)** High risk EC; **c)** Non-endometrioid EC. Selecting topics via LDA (**Middle and Right panels**): To identify differentially abundant topics between recurrent and non-recurrent cohorts (**middle panels**) we used LDA analysis. Selected in blue are those topics with negative log2 fold changes and FDR corrected p-values < 0.05: **a)** Low risk EC, topic 13; **b)** High risk EC, topics 10,16; **c)** Non-endometrioid EC, topic 1,5. In the **right panel** we depict per-topic-species probabilities matrix to examine which genus have the highest probabilities of assignment to this topic/community: **a)** Low risk EC, topic #13; **b)** High risk EC, topic #10 (with highest and lowest log2 fold changes); **c)** Non-endometrioid EC, topic #1. Given the incomplete overlap of variables between datasets, we retrained recurrence prediction models in the ORIEN cohort using only features available in TCGA to enable external validation. As in prior analyses, models were trained and validated using *MATLAB* (ML) and *TensorFlow* (DL) approaches, with oversampling applied to address class imbalance. The TCGA cohort was then used as an independent external test set (Figure 7). Although overall model performance was reduced relative to internal testing (Figure 5), reflecting the loss of informative variables, the AUCs obtained in TCGA testing fell within the 95% confidence intervals of the newly trained ORIEN models, indicating no statistically meaningful performance degradation.

We next performed topic modeling in TCGA using all features overlapping with the ORIEN-derived models (Figure 6). Despite missing variables, microbiome-related components in TCGA topic models demonstrated similar probability distributions to those observed in ORIEN, with overlapping 95% confidence intervals (**Supplementary Figure 9**). Two exceptions were noted: *Bacillus* exhibited a higher probability in TCGA compared with ORIEN (86% vs. 4%; **Supplementary Figure 9b**), whereas *Escherichia* also showed increased probability in TCGA (0.3% vs. 8%; **Supplementary Figure 9c**).

**Figure 7:**
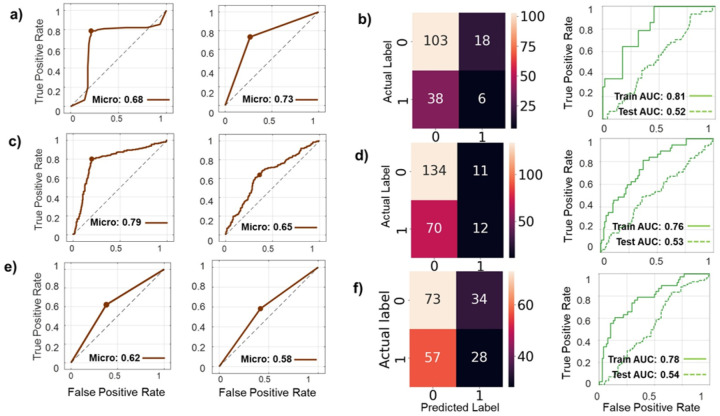
Training and validation of prediction models of EC recurrence using components of significant topics from the ORIEN analyses tested in TCGA dataset. **a)** Training and validation of a low-risk EC recurrence model in ORIEN database using *MatLab* (fine tree) and components from the significant topics microbiome, genome and immunocompetent cell invasion data (**left panel**): ROC curve AUC 0.68 (95% CI 0.55–0.81) and testing the same model in TCGA low-risk database (**right panel**): ROC curve micro AUC 0.73 (95% CI 0.65–0.81). **b)** Training and validation of a low-risk EC recurrence model in ORIEN database using *TensorFlow* (modified tutorial: Classification on imbalanced data) components from the significant topics microbiome, genome and immunocompetent cell invasion data (**left panel**): model testing confusion matrix; and testing the same model in TCGA low-risk database (**right panel**): training and testing ROC curves (0.81 [0.70–91] and 0.52 [0.30–0.76]). **c)** Training and validation of a high-risk EC recurrence model in ORIEN database using *MatLab* (efficient logistic regression) and components from the significant topics microbiome, genome and immunocompetent cell invasion data (**left panel**): ROC curve micro-AUC 0.79 (95% CI 0.69–0.88) and testing the same model in TCGA high-risk database (**right panel**): ROC curve micro-AUC 0.65 (95% CI 0.57–0.72). **d)** Training and validation of a high-risk EC recurrence model in ORIEN database using *TensorFlow* components from the significant topics microbiome, genome and immunocompetent cell invasion data **(left panel**): model testing confusion matrix; and testing the same model in TCGA high-risk database (right panel): training and testing ROC curves (0.79 [0.69–0.86] and 0.53 [0.36–0.70]). **e)** Training and validation of a non-endometrioid EC recurrence model in ORIEN database using *MatLab* (fine KNN) and components from the significant topics microbiome, genome and immunocompetent cell invasion data (**left panel**): ROC curve micro-AUC 0.62 (95% CI 0.49–0.75) and testing the same model in TCGA non-endometrioid database (**right panel**): ROC curve micro-AUC 0.58 (95% CI 0.45–0.70). **f)** Training and validation of a non-endometrioid EC recurrence model in ORIEN database using *TensorFlow* components from the significant topics microbiome, genome and immunocompetent cell invasion data **(left panel**): model testing confusion matrix; and testing the same model in TCGA non-endometrioid database (**right panel**): training and testing ROC curves (0.78 [0.61–0.94] and 0.54 [0.37–0.71]).

Given the incomplete overlap of variables between datasets, we retrained recurrence prediction models in the ORIEN cohort using only features available in TCGA to enable external validation. As in prior analyses, models were trained and validated using *MATLAB* (ML) and *TensorFlow* (DL) approaches, with oversampling applied to address class imbalance. The TCGA cohort was then used as an independent external test set (Figure 7). Although overall model performance was reduced relative to internal testing (Figure 5), reflecting the loss of informative variables, the AUCs obtained in TCGA testing fell within the 95% confidence intervals of the newly trained ORIEN models, indicating no statistically meaningful performance degradation.

Finally, Shapley value analysis was applied to both the ORIEN-trained models and TCGA-tested models to assess predictor importance (**Supplementary Figure 10**). In both low-risk and high-risk EC groups, the most influential contributors were concordant between training and testing models: *Bacillus* and *Escherichia* in low-risk EC (**Supplementary Figures 10a** and **10d**), and *SMOC1* (*ENSG00000198732*), *ENSG00000214776* pseudogene expression and *Acinetobacter* in high-risk EC (**Supplementary Figures 10b** and **10e**). In non-endometrioid EC, multiple predictors contribute consistently across training and testing models, including T Cells, CD8+ T Cells, CNVs involving *ADA* and *KRT9* genes, and microbial features such as *Bacillus* and *Escherichia* (**Supplementary Figure 10c** and **10f**).

## DISCUSSION

Endometrial cancer (EC) recurrence is a complex, multifactorial process that cannot be fully explained by tumor-intrinsic features alone. In this study, we applied an integrative, systems-level framework to model EC recurrence as an emergent property of interactions among clinical factors, tumor genomics, immune contexture, tumor-associated microbial communities, and environmental exposures. Using topic modeling to capture coordinated, cross-domain patterns and machine learning approaches to evaluate predictive performance, we identified reproducible, risk group-specific recurrence signatures that generalized across analytical platforms and independent datasets. Importantly, features selected through topic modeling retained strong predictive value in recurrence models, supporting the biological and clinical relevance of these integrated patterns. Together, these findings underscore the multifactorial nature of EC recurrence and demonstrate the feasibility of integrated, multi-domain modeling to interrogate recurrence biology at scale.

### What This Study Adds

A central finding of this study is that incorporation of environmental and neighborhood-based exposures reshaped recurrence-associated topic composition across all risk groups. Microbial communities that were prominent in models incorporating only tumor-intrinsic features were attenuated or absent after inclusion of air pollution and social–environmental variables, indicating that recurrence-associated bacterial signatures are strongly context-dependent. These findings suggest that tumor-associated microbial signals reflect broader tumor–host–environment interactions rather than static or isolated microbial effects.

### Tumor-Microbiome Interactions in EC Recurrence

Across all risk groups, *Bacillus* emerged as the bacterial genus with the highest per-topic probability, although its directionality differed by risk category. Decreased representation of Bacillus was associated with recurrence in low-risk EC, whereas increased representation was linked to recurrence in high-risk and non-endometrioid disease. This bidirectional association suggests that tumor-associated bacterial signals may reflect underlying tumor biology, host factors, or treatment context rather than uniform oncogenic or protective effects. Similar context-dependent microbial associations have been reported in other hormonally influenced malignancies,^42^ supporting the interpretation of these signals as ecological markers of tumor state.

In the low-risk EC group, microbes that were quite abundant in models before introducing environmental factors were scarcely seen afterwards, like genera *Thermothielavioides*, *Theileria*, *Rhizoctonia*, *Malassezia*, and *Candida*. It is difficult to know exactly the reason for this change in microbiome composition, because our study design cannot infer causality only association, but this is an intriguing observation that needs further follow up.

Importantly, bacterial communities identified in this study were inferred from tumor-derived bulk RNA sequencing data rather than from dedicated microbiome sampling. As such, these findings should be interpreted as relative, comparative signals reflecting tumor-associated microbial nucleic acids rather than direct measures of viable or mucosal microbiota. Nevertheless, consistent identification of specific genera across modeling approaches, risk strata, and external validation supports their relevance as ecological markers of tumor–host–environment interactions rather than isolated microbial drivers. These results should therefore be viewed as hypothesis-generating.

### Social and Environmental Determinants Associated with EC Recurrence

Environmental exposures and social determinants of health emerged as consistent modifiers of recurrence-associated patterns across EC risk groups. Ozone (O_3_) was repeatedly identified as a component of recurrence-associated topics in all three risk strata, supporting a biologically plausible link between ambient oxidative stress and EC recurrence. O_3_ exposure has been implicated in oxidative DNA damage, inflammatory signaling, immune modulation, and estrogen dysregulation, pathways central to EC pathogenesis and progression, particularly in hormonally responsive tissues.^36–45^ Although individual-level exposure assessment was not feasible, the reproducible association of O_3_ with recurrence-associated topics suggests that environmental oxidative stress may act as a contextual modifier of tumor biology rather than an isolated risk factor.

In parallel, social and environmental determinants of health contributed to the composition of recurrence-associated topics, most prominently in high-risk and non-endometrioid EC. Features such as proximity to high-volume roadways and airports, impaired water bodies, and limited food access, proxies for structural and environmental disadvantage, were among the variables influencing these patterns. These findings are consistent with growing evidence linking neighborhood-level exposures to cancer outcomes and support a model in which place-based factors shape tumor biology through indirect, cumulative mechanisms.^46,47^ Notably, these associations persisted despite individual-level race or ethnicity not emerging as dominant predictors, underscoring the potential importance of structural context beyond individual demographic characteristics.

### Risk Group–Specific Biological Programs Underlying EC recurrence.

Recurrence-associated patterns differed substantially by clinical risk group, reinforcing the biological heterogeneity of EC recurrence pathways and arguing against a single, unified mechanism of relapse. Low-risk endometrioid EC recurrence was driven predominantly by metabolic and microbiome-associated features, with minimal persistence of clinical variables beyond body mass index (BMI). In contrast, high-risk and non-endometrioid tumors were characterized by greater contributions from gene expression programs, immune contexture, stromal activation, and environmental domains. This stratified behavior supports the concept that recurrence mechanisms, and therefore opportunities for refined risk stratification or intervention, may differ fundamentally across EC subtypes.

Obesity, and its proxy BMI, are intrinsically linked to estrogen metabolism, EC pathogenesis, metabolic syndrome, and microbiome dysbiosis.^36^ Accordingly, the persistence of BMI as a component of low-risk recurrence models is biologically plausible, particularly given the estrogen-responsive nature of low-risk endometrioid tumors. In this group, recurrence-associated patterns reflected a coordinated imbalance involving reduced *Bacillus*, elevated ozone exposure, copy number alterations in genes primarily related to nucleocytoplasmic transport, and increased CAF representation, suggesting a convergence of hormonal, metabolic, microbial, and stromal influences that may favor tumor re-emergence.

In contrast, clinical variables previously associated with recurrence risk in earlier analyses, including ethnicity, chemotherapy exposure, albumin, and red blood cell distribution width, did not persist within the integrated topic models for high-risk or non-endometrioid EC. Instead, recurrence in these groups was characterized by dysregulation of gene, pseudogene, and isoform expression involving T-cell signaling pathways, lipid and carbohydrate metabolism, folate transport and metabolism, and basal transcriptional machinery. These molecular programs co-occurred with pronounced immune and stromal features, including increased CAF abundance and macrophages M1 infiltration, as well as consistent microbiome dysbiosis marked by increased *Bacillus* and *Candida* and decreased *Escherichia*. Elevated ozone exposure was again observed, suggesting a recurring environmental backdrop across higher-risk disease.

Notably, increased CAF representation emerged as a shared feature across all EC subtypes associated with recurrence, consistent with prior evidence implicating stromal remodeling in disease progression.^48^ However, heightened macrophages M1 infiltration was restricted to high-risk and non-endometrioid tumors, underscoring risk group–specific immune dynamics. Together, these findings highlight that EC recurrence arises from distinct, subtype-dependent biological programs shaped by interacting tumor-intrinsic, microenvironmental, microbial, and environmental factors, rather than from a uniform recurrence pathway.

### Clinical and Translational Implications of Integrated Recurrence Modeling

This study was not designed to produce an immediately deployable clinical prediction tool. Rather, it establishes a scalable, modular analytic framework for integrating heterogeneous biological and environmental data to interrogate EC recurrence biology at a systems level. Although the recurrence prediction models developed here performed comparably to previously published models, they consistently demonstrated that features emerging from integrated topic models encompassing clinical variables, tumor genomics, immune contexture, microbiome composition, and environmental exposures capture biologically meaningful recurrence-associated patterns. Notably, incorporation of air pollution variables altered microbiome feature composition without degrading model performance, underscoring the tightly interconnected nature of tumor, host, microbial, and environmental factors influencing EC relapse.

To minimize overfitting and assess generalizability, both topic models and recurrence prediction models were evaluated using the TCGA EC cohort as an independent external dataset. Because TCGA lacks several key variables present in ORIEN, including environmental exposures such as air pollutants, models were retrained in ORIEN using TCGA-compatible features prior to external testing. Despite these constraints, model performance in TCGA remained within the 95% confidence intervals of the retrained ORIEN models, indicating preserved predictive stability. Differences in cohort composition and data structure likely influenced external validation performance, including earlier-era sample collection in TCGA, more limited follow-up and case status reporting, and reduced histologic diversity within non-endometrioid tumors compared with ORIEN. These limitations highlight the challenges of external validation for integrated, multi-domain models and emphasize the importance of contemporary, deeply annotated cohorts for translational modeling.

From an NIH translational research perspective, this work primarily occupies the T0–T1 space, generating integrated biological insights and analytically validated recurrence signatures rather than clinical decision tools. Importantly, however, it provides a foundation for progression toward T2 translation. Specifically, this framework enables prospective cohort studies incorporating longitudinal biospecimen collection, spatially resolved tumor and microenvironment profiling, and microbiome-specific assays to validate and refine recurrence-associated programs. Such studies can inform risk-adapted surveillance strategies, identify biologically defined subgroups most likely to benefit from targeted interventions, and guide the rational design of prevention or interception trials. By establishing a reproducible, extensible modeling architecture, this study advances the field toward clinically actionable integration of tumor biology, host context, and environmental exposures in EC recurrence.

### Strengths

A major strength of this study is the integration of diverse data modalities within a unified analytical framework. By jointly modeling clinical, pathological, genomic, immune, microbiome, and environmental features, we move beyond traditional single-domain analyses and provide a more holistic view of EC recurrence biology. Topic modeling enabled identification of coordinated feature sets that reflect biologically meaningful processes rather than isolated variables, while subsequent machine learning models demonstrated that these topic-derived features are also robust predictors of recurrence across multiple risk groups.

Another key strength is the use of complementary analytical platforms. Consistent performance across MATLAB-based machine learning and TensorFlow-based deep learning approaches supports the robustness of our findings and reduces the likelihood that results are driven by platform-specific modeling assumptions. The application of Shapley value analysis further strengthens interpretability by clarifying the relative contribution of individual predictors within the best-performing models, an important consideration for translational relevance.

External validation using the TCGA endometrial cancer cohort represents an additional strength. Despite incomplete overlap of features and known limitations of TCGA data, recurrence models trained in ORIEN and tested in TCGA demonstrated performance that remained within the confidence bounds of internally validated models. Concordance of key predictors—particularly microbiome-associated features, immune cell populations, and select genomic alterations—between ORIEN training models and TCGA testing models provides evidence of generalizability and biological consistency across independent datasets.

### Limitations

Several limitations should be acknowledged. First, tumor-associated microbial signals were inferred from bulk RNA sequencing rather than from dedicated microbiome sequencing platforms. As such, these findings should be interpreted as relative, comparative signals reflecting microbial nucleic acids present in tumor-derived data rather than direct measures of viable or mucosal microbiota. While consistent identification of specific genera across risk groups, modeling strategies, and external validation supports their relevance as ecological markers, functional and spatial validation will be required to clarify causal relationships.

Second, integration of environmental exposures was constrained by data availability. County-level identifiers were required to link air pollution and social or environmental determinants of health to individual patients, resulting in reduced sample sizes for models incorporating these variables. This limitation likely attenuated model performance in some settings and may have reduced power to detect stronger environmental effects. Moreover, TCGA lacks county-level identifiers entirely, precluding external validation of environmental features and limiting assessment of their generalizability.

Third, external validation using TCGA was affected by incomplete overlap of features between datasets, differences in follow-up duration, case status reporting, and timing of biospecimen collection. These factors necessitated retraining of recurrence models in ORIEN using TCGA-compatible features and likely contributed to reduced absolute performance in external testing. Nevertheless, the observation that TCGA testing performance remained within the confidence intervals of retrained ORIEN models supports the stability of the underlying predictive framework despite these constraints.

## CONCLUSION

In summary, endometrial cancer recurrence emerges from this analysis as an emergent property of coordinated interactions among tumor-intrinsic programs and extrinsic contextual factors, rather than as the consequence of any single biological domain. By integrating clinical, pathological, genomic, immune, microbiome, and environmental features within a systems-level modeling framework, we demonstrate that these complex interactions can be quantified, interpreted, and externally validated across independent cohorts.

Importantly, both intrinsic tumor and host characteristics and extrinsic environmental and social exposures contributed to recurrence-associated patterns, with their relative influence varying by clinical risk group. These findings underscore the biological heterogeneity underlying EC relapse and highlight the limitations of one-size-fits-all prediction approaches. Collectively, this work supports the need for more individualized, context-aware models of disease outcomes and establishes an extensible analytic foundation for future translational efforts aimed at improving EC recurrence risk stratification, prevention, and intervention.

## Supplementary Material

Supplementary Files

This is a list of supplementary files associated with this preprint. Click to download.

- Supplementarymaterial12726NPJPO.docx
- SupplementaryTable5.xlsx

## Data Availability

The data used in this study was generated through private funding by Aster Insights (www.asterinsights.com) in collaboration with the Oncology Research Information Exchange Network (ORIEN, www.oriencancer.org). Inquiries regarding access to the data or collaboration within ORIEN should be submitted here at https://researchdatarequest.orienavatar.com/. For non-ORIEN academic researchers, only processed data outputs from clinical, whole exome and whole transcriptome data may be available, where applicable.
